# Pathogenic Mechanism of Autoimmune Diabetes Mellitus in Humans: Potential Role of Streptozotocin-Induced Selective Autoimmunity against Human Islet *β*-Cells

**DOI:** 10.3390/cells11030492

**Published:** 2022-01-31

**Authors:** Bao Ting Zhu

**Affiliations:** 1 Shenzhen Key Laboratory of Steroid Drug Discovery and Development, School of Medicine, The Chinese University of Hong Kong, Shenzhen 518172, China; BTZhu@CUHK.edu.cn; 2 Department of Pharmacology, Toxicology and Therapeutics, School of Medicine, University of Kansas Medical Center, Kansas City, KS 66160, USA

**Keywords:** autoimmune diabetes, pathogenic mechanism, streptozotocin-induced *β*-cell damage

## Abstract

Human type 1 diabetes mellitus is a chronic autoimmune disease characterized by the selective loss of insulin-producing *β*-cells in pancreatic islets of genetically susceptible individuals. In this communication, a new hypothesis is postulated which is based on the observations that streptozotocin (STZ), a chemically reactive and cytotoxic compound produced by certain gram-positive bacteria, can be preferentially taken up into islet *β*-cells and induce cytotoxicity and autoimmunity. It is hypothesized that humans might be occasionally exposed to STZ through opportunistic infections with the STZ-producing bacteria and/or through ingestion of certain food products that contain STZ. In addition, the potential presence of the STZ-producing bacteria in the gut microbiota of some individuals might be another source of long-term STZ exposure. Because of the high chemical reactivity of STZ and its breakdown products, these chemicals can covalently modify certain cellular macromolecules (e.g., DNA and proteins), and the covalently modified cellular components would serve as new antigens, potentially capable of inducing both humoral and cellular autoimmune responses in the islets of certain individuals. In addition to STZ exposure, the eventual development of autoimmunity against STZ-exposed islet *β*-cells also depends critically on the genetic predisposition of the susceptible individuals plus the opportunistic presence of a conducive, strong environmental trigger, which often is presented as severe febrile viral infections subsequently inducing strong aberrant reactions of the body’s immune system. The proposed pathogenic hypothesis is supported by a considerable body of direct and indirect evidence from laboratory animal studies and clinical observations. Certainly, more experimental and clinical studies are needed to carefully further examine each of the key components of the proposed pathogenic hypothesis.

## 1. Introduction

Clinically, there are four main types of diabetes mellitus (DM) in humans, namely, type 1 DM (T1DM), type 2 DM, gestational DM, and other specific types of DM [1]. T1DM is characterized by the loss of insulin-producing *β*-cells in the islets of Langerhans in the pancreas, resulting in insulin deficiency. While T1DM can be further classified as autoimmune-mediated or idiopathic, the majority is thought to be immunologically mediated, in which islet *β*-cell loss is the result of a selective autoimmune attack in genetically susceptible individuals [2,3,4]. There is no known preventive measure available at present for T1DM. Most affected people are otherwise healthy and of a healthy body weight when disease onset occurs [4]. Sensitivity and responsiveness to insulin are usually normal, especially in the early stages [4]. T1DM can affect both children and adults but was traditionally termed “juvenile diabetes” because the majority of cases occur in children [4]. Type 2 DM mainly results from insulin resistance, a condition in which cells in the body (such as hepatocytes, skeletal muscle cells, and adipocytes) fail to respond to insulin properly [5]. Gestational DM occurs when pregnant women without a previous diagnosis of DM develop hyperglycemia [6]; this condition sometimes may precede the onset of type 2 DM [7]. There are also certain “specific types” of DM in humans which are due to other causes, such as monogenic diabetes syndromes and diseases of the exocrine pancreas [8].

The pathogenic mechanism underlying the autoimmune attack in T1DM is still poorly understood at present. In 1986, a disease model for the development of autoimmune T1DM was proposed by Eisenbarth [9]. It was suggested that people are born with various degrees of susceptibility to developing T1DM. When exposed to certain environmental risk factors, it is likely to trigger autoimmunity and destruction of islet *β*-cells in susceptible individuals, which subsequently results in the development of T1DM [9]. Over the years, a long list of environmental risk factors has been suspected of playing a role in islet autoimmunity and the subsequent development of T1DM, which include viruses, dietary factors, vitamin D, nitrites and *N*-nitroso compounds, gluten and fiber, vaccinations, certain pollutants and toxins, microbiomes, perinatal and psychological stress, and others [10].

In this communication, a new hypothesis is proposed, which speculates that infections caused by streptozotocin (STZ)-producing bacteria coupled with the body’s aberrant immune response, which often is triggered by the accompanying febrile viral infections, are an important pathogenic cause for human autoimmune T1DM in genetically susceptible individuals.

## 2. Hypothesis

STZ is produced by certain strains of the gram-positive bacteria *Streptomyces achromogenes*, that have a wide distribution in the environment [11,12,13,14,15]. It was originally identified in the late 1950s as an antibiotic [11]. Later, it was found that STZ has preferential cytotoxicity in the insulin-producing islet *β*-cells [16,17]. Because of this unique pharmacological property, STZ has been used for treating carcinoma of human pancreatic islet cells [18,19]. Regarding the mechanism of STZ’s anticancer action, it was suggested that STZ, being a glucosamine–nitrosourea compound, can induce strong cytotoxicity mostly by causing damage to cellular DNA and proteins through covalent modifications, although other mechanisms were also thought to be involved [20].

**Figure 1:**
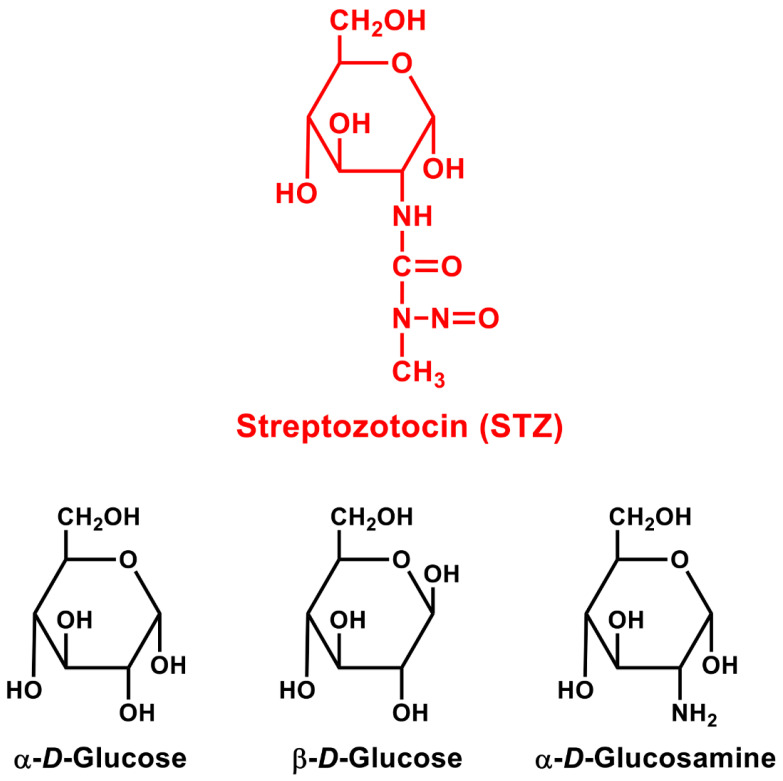
The chemical structure of STZ in comparison with *α*-glucose, *β*-glucose, and *α*-*D*-glucosamine. STZ contains an *α*-glucose chemical moiety, which is believed to be the structural basis for its ability to serve as a substrate for the glucose transporter GLUT2. It is of note that STZ also shares a close structural similarity to *α*-*D*-glucosamine.

Structurally, STZ is similar to glucose (Figure 1) and is selectively transported into islet *β*-cells by GLUT2 (a glucose transporter present in the cell membrane of islet *β*-cells), but it is basically not recognized by other glucose transporters [16,17]. Because the islet *β*-cells of some laboratory animals (e.g., rats and mice) express very high levels of GLUT2 and thus are preferentially sensitive to STZ’s cytotoxicity [16,17], STZ has been commonly used as a tool of choice in biomedical research to produce animal models of DM through selective destruction of their islet *β*-cells [21,22,23,24]. Here it is of note that while the precise mechanism by which STZ induces autoimmune-type DM in animal models is still not completely clear at present, there is, nonetheless, considerable experimental evidence clearly demonstrating that administration of multiple low doses of STZ (MLD-STZ) could produce a long-lasting autoimmune-type DM in rodents with pathogenic characteristics closely resembling those of human autoimmune T1DM (discussed in detail later).

**Figure 2:**
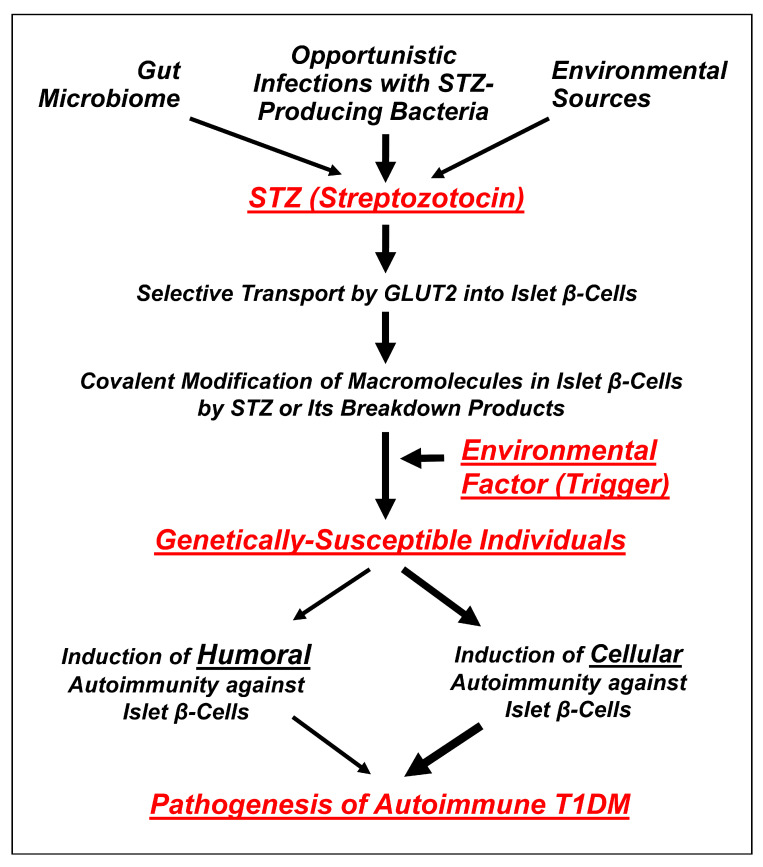
A proposed hypothesis on the potential role of STZ in the pathogenesis of autoimmune T1DM in humans. A detailed description of the hypothesis is provided in the main text under Section 2 (“Hypothesis”).

**Figure 3:**
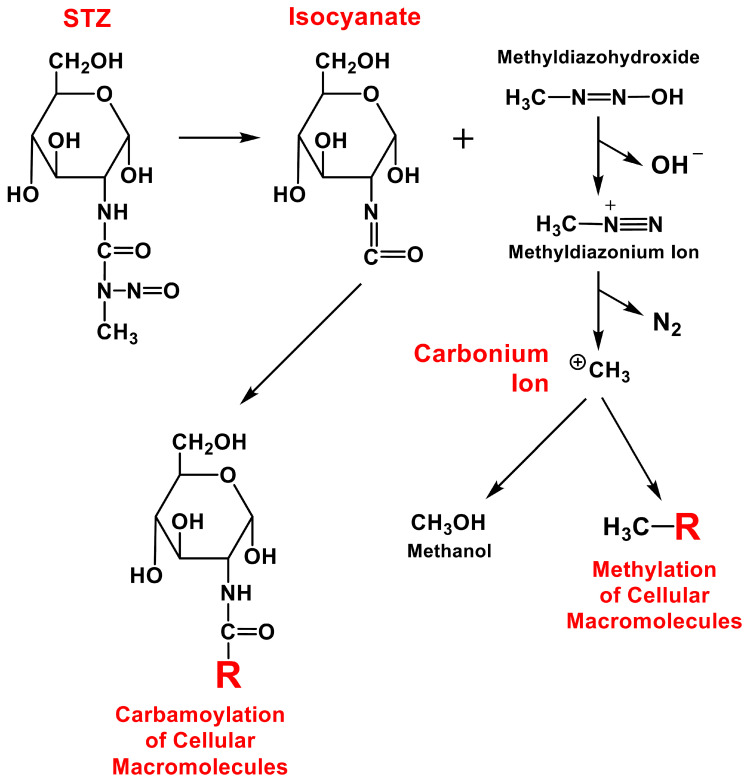
Pathways of chemical decomposition of STZ in pancreatic islet *β*-cells and the covalent modifications of cellular macromolecules (mostly proteins and DNA) by chemically reactive decomposition products of STZ (e.g., isocyanate and carbonium ion). While the carbonium ion is highly reactive and can covalently modify (methylate) various macromolecules within islet *β*-cells in a facile manner, STZ’s isocyanate derivative is bulkier and likely more antigenic once it covalently modifies a macromolecule (such as a protein) through carbomoylation.

**Figure 4:**
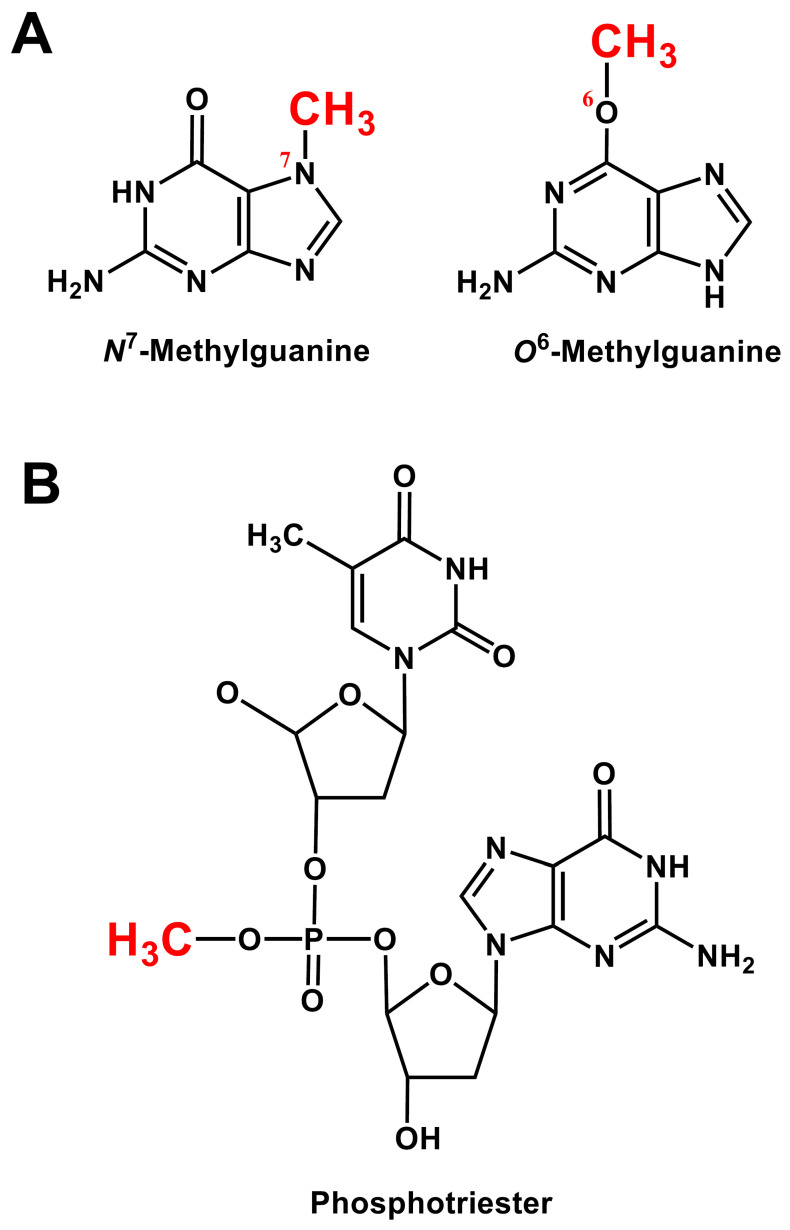
Methylation by the STZ breakdown product carbonium ion of the guanine bases contained in cellular DNA and RNA at the *N*^7^ and *O*^6^ positions (**panel A**) and of the DNA’s phosphate backbone (**panel B**).

In this paper, a hypothesis is proposed that attempts to explain the potential contribution of STZ in the etiology of human autoimmune T1DM (Figure 2). Specifically, it is hypothesized that humans might occasionally be exposed to STZ through opportunistic infections with the STZ-producing bacteria (e.g., *Streptomyces achromogenes*) or through certain food products that contain STZ or drinking contaminated water. In addition, the possible presence of the STZ-producing bacteria in human gut microbiota (discussed later) may also increase the likelihood of a chronic STZ exposure. Moreover, it is further hypothesized that STZ generated by the infecting bacteria may preferentially accumulate in pancreatic islet *β*-cells and subsequently cause chemical damage to these cells through covalent modifications of their cellular macromolecules (such as DNA and proteins). It is speculated that the direct chemical toxicity to islet *β*-cells caused by relatively lower doses of STZ (which would not quickly kill all or most islet *β*-cells) usually would be short lasting and likely would not cause permanent DM as the remaining islet *β*-cells are expected to slowly regenerate and thus recover, at least partially, from the initial chemical damage. This suggestion appears to be in line with an earlier clinical study [25] showing that treatment of domestic pigs with STZ at high doses (100–150 mg/kg body weight) would produce a complete and permanent diabetic condition, but when the animals were treated with a slightly lower dose of STZ (85 mg/kg body weight), they would only develop a transient diabetic reaction, suggesting that the chemically damaged islet *β*-cells might be able to regenerate and partially recover from the initial damage. However, because STZ can also covalently modify cellular components in islet *β*-cells, the covalent modifications of certain cellular macromolecules (such as DNA and proteins) by STZ and its chemically reactive breakdown products (Figure 3 and Figure 4) may lead to the formation of new cellular autoantigens. These changes might trigger the production of autoimmune-type humoral and cellular immune responses, and subsequently, initiate long-lasting autoimmune attacks against islet *β*-cells. It is expected that in most cases, the induced autoimmune T and and B cells would only attack the islet *β*-cells that contain antigenic cellular macromolecules covalently modified by STZ or its chemically reactive breakdown products. Here it should be noted that in some cases it is also possible that the autoimmunity induced in the body following exposure to STZ-modified antigenic cellular macromolecules might cross-react with the same cellular components of islet *β*-cells even in the absence of covalent modifications.

While the appearance of autoantibodies against islet *β*-cells is widely viewed as the first detectable sign of *β*-cell autoimmunity, it is the cellular immune response against STZ-modified islet autoantigens that eventually causes persistent *β*-cell damage and the pathogenesis of human T1DM [26]. It is speculated that cellular, as well as humoral immune responses, are both driven by STZ-modified islet autoantigens in genetically susceptible individuals under conducive conditions triggered by environmental factors.

In addition to STZ exposure and the formation of covalently modified new cellular autoantigens, it should be clearly pointed out that the eventual development of the autoimmune-type responses in the islets of certain individuals would also depend critically on the genetic predisposition of the susceptible individuals plus the timely presence of an environmental trigger, which might be in the form of aberrant strong immune responses of the body occurring concurrently (such as in response to severe febrile viral infections). It is speculated that when these critical genetic and environmental factors are absent, pathogenic autoimmune responses would not take place even if the STZ-exposed islet *β*-cells have already formed new cellular autoantigens.

Provided below (Section 3, Section 4, Section 5, Section 6, Section 7, Section 8 and Section 9) is a critical evaluation of each major component of the proposed hypothesis along with a discussion of the available experimental and clinical observations that may offer direct, indirect, or circumstantial support for the proposed hypothesis.

## 3. Potential Sources of Human STZ Exposure

### 3.1. STZ-Producing Bacteria

STZ is produced by certain strains of *Streptomyces achromogenes* [11,12,13,14,15] and has a broad-spectrum antibiotic activity and selective antineoplastic property (reviewed in [13,20]). *Streptomyces achromogenes* belongs to the genus *Streptomyces* which are gram-positive bacteria with a wide distribution and abundant presence in soil [13,14,15,27]. In addition to soil, habitats of *Streptomyces* also include animal fodders and other organic materials [15].

### 3.2. Gut Microbiota

It is known that there are about 500 to 1000 species of gut microorganisms present in humans [28]. Studies have clearly indicated that gut microbiota is associated with the pathogenesis of T1DM [29,30,31]. It was reported earlier that modulation of gut microbiota in Bio-Breeding DM-prone rats, a rat model for T1DM, through antibiotic use results in decreased incidence and delayed onset of T1DM [29]. Mechanistically, it is possible that certain strains of the STZ-producing bacteria might be present in the gut microbiota of some susceptible human subjects, thereby contributing to an increased risk for developing T1DM. Here it should be noted that it was speculated earlier that there might be certain degrees of molecular mimicry between islet cell antigens and certain microorganism components, which might also partially contribute to the pathogenesis of T1DM [32].

In addition, it is speculated that livestock, such as cows, may be fed with fodders containing STZ-producing *Streptomyces* as well as the toxic chemical STZ. After entering the body of cows, the STZ-producing *Streptomyces* might become part of cows’ gut microbiota, and as a result, STZ might be present in cow’s meat and milk either in its free form or in covalently adducted forms (i.e., STZ and its breakdown products may covalently modify macromolecules of cow meat and milk). When humans ingest cow’s meat or milk as food, STZ may enter the human body. Regarding this potential route of STZ exposure in humans, there were some epidemiological studies speculating that early dietary intake of customary cow’s milk formula under the age of 5 might be associated with an increased risk of *β*-cell autoimmunity and T1DM [33,34]. A more detailed discussion on this highly speculative and controversial topic is provided later in Section 9.

### 3.3. Environmental Sources of STZ

It was reported that soil *Streptomyces* can readily contaminate rivers and freshwater lakes after heavy rainfalls; thus the drinking water supplies sometimes may become contaminated with *Streptomyces* [14]. Under such circumstances, humans and animals are likely to be occasionally exposed to low levels of *Streptomyces* and their toxic product STZ through the water they drink.

### 3.4. Opportunistic Infections with STZ-Producing Bacteria

Generally, *Streptomyces* are usually considered of low pathogenicity in humans. While the reported cases of infections caused by *Streptomyces* are relatively small compared to many other bacteria, it has also been noted that the real cases of human infection with *Streptomyces* may be greatly underestimated [13,14]. Mycetoma, the chronic suppurative infection of the skin and underlying soft tissue and bone, is a more common presentation of *Streptomyces* infection [35]. Moreover, there are also reported cases of *Streptomyces* infections that lead to infections in the lung, blood stream, and/or pericardia in the patients [14,35,36,37,38,39]. Based on existing clinical case reports, *Streptomyces* infections often occur in individuals with underlying diseases that would weaken the body’s immune system functions. An earlier paper reviewing 19 cases of invasive *Streptomyces* infections noted that some of the preexisting conditions, such as cancer, AIDs, autoimmune disease, presence of a central venous catheter, and prosthetic heart valve, were found in most cases (14/19) [37,38].

These findings support a general view that when the body’s immune system function is compromised, opportunistic infections with STZ-producing *Streptomyces* may take place. It is further speculated that when the body’s immune system function is under severe stress caused by the accompanying febrile viral infections, STZ, which is produced by the infecting *Streptomyces*, might induce aberrant autoimmune responses in pancreatic islets of genetically susceptible individuals, which results from STZ’s covalent modifications of the cellular components in islet *β*-cells. The detailed mechanism by which STZ induces islet *β*-cell damage and the ensuing autoimmune responses are described in detail below in Section 4 and Section 5.

## 4. STZ Can Readily Induce Autoimmune Diabetes in Animal Models

STZ is a naturally occurring alkylating agent. Structurally, it is nitrosamide methylnitrosourea (MNU) linked to the C-2 position of *α*-*D*-glucose [40] (Figure 1). The glucose moiety is a component in STZ that determines its specific cytotoxicity in islet *β*-cells, as these cells preferentially express the glucose transporter protein GLUT2 [7,8], which has a high affinity for the transport of STZ. Studies using ^14^C-labeled STZ found that STZ is preferentially taken up by islet *β*-cells [30]. Consistent with this observation, STZ has markedly higher toxicity in islet *β*-cells than MNU, a well-known alkylating agent [41].

Because STZ can selectively destroy islet *β*-cells [42,43], it has been widely used in diabetes research as a tool to induce hyperglycemia and insulitis in animal models [21,22,23,24]. In general, rats are sensitive to the effect of STZ and therefore are among the commonly used animal models in research settings [21,22,23,24]. The intensity and duration of hyperglycemia in rats depend on the amount and frequency of STZ administration. Based on many experimental observations from different research groups, there are two well-characterized regimens of STZ administration that are best suited to induce diabetic conditions in rats [21]. One is the “single high-dose regimen”, which gives a single injection of a high dose of STZ (usually at 200 mg/kg body weight) in rats [21]. At high doses, it has been suggested that STZ could induce GSH depletion, severe oxidative stress, and cell death in pancreatic islet β-cells in rats [44,45], which was accompanied by rapid elevation of blood glucose levels usually within a very short time (around 48 h) [21]. This model has been widely used as a short-term DM rodent model, and diabetic animals are usually observed and tested for a relatively short experimental period [21]. Another method is commonly referred to as the “multiple low-dose STZ (MLD-STZ) regimen”, which tends to induce immune responses in the pancreatic islets of treated animals [21,22,23,24]. It was found that the experimental animals (usually rats and mice) that were treated with MLD-STZ (usually at 40 mg/kg body weight once daily for five consecutive days) would develop insulitis and complex immune responses in the islets after a latent period of about five days after the last dose of STZ, and these changes were accompanied by hyperglycemia and markedly reduced insulin biosynthesis and secretion from islet *β*-cells [21,22,24,44,45,46]. Although in most studies, the pathogenesis and symptoms of DM were only observed for a relatively short period of time (up to several weeks), there were also studies that explored the long-term effect of MLD-STZ administration in animals. For instance, it was found that MLD-STZ administration resulted in stable and long-lasting hyperglycemia in rats with no insulin response to glucose administration and significant morphological derangements in the islets 3 months after receiving MLD-STZ treatment [47]. Similar findings were also reported that the glucose levels were significantly elevated in rats 16 weeks following MLD-STZ administration [48]. Together, these results suggest that the MLD-STZ regimen is capable of inducing a stable, long-lasting DM in experimental animals.

There were also studies showing that the MLD-STZ regimen could induce the kind of autoimmune responses in animals that closely resemble the characteristic immunological changes commonly seen in patients with T1DM [49]. A more detailed discussion of the literature evidence on this particular subject is provided in Section 6 and Section 7.

Here it should be noted that there were some earlier studies showing that the MLD-STZ regimen induced mostly direct pancreatic islet damage instead of autoimmune-type responses in islet *β*-cells [46]. This discrepancy might be due to the fact that some animals under certain conditions (such as starvation) became especially sensitive to the cytotoxic effect of MLD-STZ in their islet *β*-cells. If islet *β*-cells of animals become too sensitive to the direct toxicity of MLD-STZ under certain conditions (which would be somewhat similar to those animals receiving multiple high doses of STZ), then the islet cells would be destroyed too quickly, and under such conditions, the animals may not have sufficient time to develop the autoimmune-type responses in the islet *β*-cells [46].

Studies have shown that different species of animals have different susceptibility to STZ toxicity in their islets [25,26,47,48,49,50,51,52,53,54,55]. Rats, mice, dogs, pigs, and monkeys appear to be highly susceptible to the diabetogenic and cytotoxic effects of STZ and have been commonly used as DM animal models [17,25,47,48,49,50,51,52,53,54]. In comparison, rabbits are relatively resistant to STZ, and higher doses of STZ are needed to trigger a similar level of diabetic response in vivo [55]. Furthermore, animals with different strains, genotypes, and gender also have significantly different sensitivity to STZ-induced islet damage [21,56], suggesting that the different genetic backgrounds of the laboratory animals may affect the susceptibility to STZ-induced autoimmune-type DM.

It is of note that while human islet *β*-cells can effectively take up STZ and thus cause cytotoxicity in these cells [18], it has also been noted that human islet *β*-cells are less sensitive to STZ than islet *β*-cells from rodents [57,58]. It appears that GLUT2, the primary glucose transporter contained in rodent islet *β*-cells which has a high affinity for transport of STZ, may play a lesser role in human islet *β*-cells [59]. It is not known whether different individuals might have varying levels of GLUT2 and other glucose transporters in their islet *β*-cells, which could be an important factor determining individual susceptibility to STZ-induced islet cytotoxicity as well as autoimmunity.

## 5. STZ Can Covalently Modify Cellular Components to Form Autoimmune Antigens

It is hypothesized that STZ can covalently modify cellular components such as DNA and proteins to form autoimmune antigens which are targeted by autoimmune T and B cells. As discussed below, there is ample evidence in support of this possibility.

### 5.1. Covalent Modifications of Cellular DNA

As an alkylating agent, STZ is known to produce a variety of DNA damages in islet cells, including covalently modified nucleobases, strand breaks, alkali-labile sites, unscheduled DNA synthesis, and chromosomal aberrations [20]. Wilson and Leiter [40] have proposed a chemical mechanism by which STZ might cause DNA damage in islet *β*-cells. It was postulated [40] that once transported inside the cell, STZ can spontaneously decompose to an isocyanate compound plus methyldiazohydroxide (Figure 3). The isocyanate component could either carbamoylate various cellular components or undergo intramolecular carbamoylation. On the other hand, the methyldiazohydroxide tends to form a highly reactive carbonium ion which could alkylate various cellular components such as DNA and proteins. The carbonium ion could react with nucleophilic centers in DNA and generate DNA lesions which are subsequently removed by excision repair. This excision repair process involves the enzyme poly (ADP-ribose) synthetase using nicotinamide adenine dinucleotide (NAD) as a substrate. It has been speculated that in islet *β*-cells, this enzyme is highly activated to such an extent that NAD might become depleted, resulting in cellular dysfunction and ultimately cell death [40]. In partial support of this suggestion, earlier studies have shown that when rodents were jointly treated with STZ and nicotinamide, the toxicity of STZ in islet *β*-cells of these animals was significantly reduced compared to animals treated with STZ alone [60,61].

In STZ-treated animals, the *N*^7^-position of guanine was found to be the most-frequently alkylated site in DNA, and the *O*^6^-position of guanine is the most-commonly alkylated base oxygen (Figure 4A). It is postulated that the *O*^6^-guanine lesion may play a critical role in the MLD-STZ model of DM because alkylation of this oxygen interferes with hydrogen bonding and allows guanine to mispair with thymine, thereby causing a point mutation. A lesion of this type may activate the expression of a repressed gene that codes for a protein not normally recognized by the immune system (e.g., a fetal protein or a retrovirus). Moreover, the repair of *O*^6^-methyl guanine is different from the repair of adducts formed with nitrogens.

In addition to the nucleobase positions, another target for STZ alkylation of DNA is the phosphate backbone which results in the formation of a phosphotriester (Figure 4B). These lesions have been reported to be only slowly repaired or not repaired at all [40]. Although the exact biological consequence of the phosphotriesters has yet to be determined, it was speculated that these lesions might aid in eliciting an autoimmune reaction against islet *β*-cells [40].

### 5.2. Covalent Modifications of Cellular Proteins

In addition to covalent modification of the DNA, many earlier studies have also investigated the ability of STZ to modify cellular proteins (Figure 3). It was shown that treatment of rats with STZ results in a rapid increase in islet *β*-cells the levels of intracellular protein modification [59]. Modification of key proteins in islet *β*-cells is thought to be an important mechanism by which MLD-STZ administration induces long-lasting cellular damages through autoimmune responses and attacks [40]. In fact, it has been suggested earlier that factors other than causing DNA damage also contribute critically to the toxicity of STZ in islet *β*-cells [62]. Experimental studies by Wilson et al. [41] showed that in islet *β*-cells following exposure to ^14^C-labeled STZ, a smaller proportion of the carbonium ion produced by STZ alkylated DNA whereas a far greater proportion of the ion actually alkylated proteins, indicating that proteins were covalently modified by STZ and its breakdown products to a far greater extent than DNA.

The individual proteins that are specifically alkylated by STZ have not yet been identified. Earlier, Wilson et al. [41] speculated that the STZ-targeted proteins might include components necessary for ATP production (such as glycolytic or mitochondrial enzymes). It is hypothesized that some of the proteins that are covalently modified by STZ might be involved in glucose transport and/or metabolism as well as insulin secretion in islet *β*-cells. Covalent modifications (mostly methylation) of certain cellular proteins may alter their conformations, subsequently resulting in the formation of new autoantigens [40].

Presently, it is not known which cellular components in islet *β*-cells that are covalently modified by STZ and its breakdown products would become pathogenic targets for autoimmune attack by activated T cells and B cells. Some of the cellular targets, such as insulin, glutamic acid decarboxylase, tyrosine phosphatase-related insulinoma-associated 2 molecules, and zinc transporter-8 [3,63] whose autoantibodies have been identified in many T1DM patients, might be the potential targets for covalent modifications by STZ. Two potential additional cellular targets that might also be covalently modified by STZ are separately discussed below.

#### 5.2.1. Possibility 1. STZ and the *O*-Glycosylation Process in Islet *β*-Cells

During *O*-glycosylation, cellular enzyme *O*-GlcNAc transferase (OGT) uses the substrate UDP-*N*-acetylglucosamine (UDP-GlcNAc) to attach a single *O*-GlcNAc to nuclear and cytosolic proteins on their serine or threonine residues. Conversely, the enzyme *O*-GlcNAc-selective *N*-acetyl-*β*-*D*-glucosaminidase (*O*-GlcNAcase) removes *O*-GlcNAc, returning the protein to its baseline state [64]. This pathway is thought to be especially important in pancreatic islet *β*-cells since they contain much higher levels of OGT than other cell types [65]. This enables islet *β*-cells to respond to physiological increases in glucose levels by converting glucose to the OGT substrate UDP-GlcNAc, thereby dynamically coupling intracellular *O*-linked protein glycosylation to the extracellular glucose levels [65]. As a result, it was speculated that islet *β*-cells are especially susceptible to disruption of the *O*-glycosylation pathway.

STZ is a UDP-GlcNAc analog that can cause *β*-cell toxicity by irreversibly inhibiting the function of *O*-GlcNAcase, likely through covalent modification [65]. Research has found that treating cultured cells with STZ during *O*-GlcNAc peptide biosynthesis results in hyperglycosylation of the peptide since STZ can inhibit the catalytic activity of *O*-GlcNAcase. It is also found that islet *β*-cells express very high levels of the mRNA encoding OGT which catalyzes cytoplasmic protein *O*-glycosylation. When STZ blocks *O*-GlcNAc removal from intracellular proteins, *β*-cells would experience a rapid accumulation of this modified protein. Accordingly, it is speculated that STZ exposure might lead to the production of autoantibodies against STZ-modified OGT in T1DM patients.

#### 5.2.2. Possibility 2. STZ May Covalently Modify GLUT2

GLUT2 is a transmembrane carrier protein that facilitates glucose movement across the plasma membrane [16,17]. Since STZ contains a glucose moiety, it is proposed that this molecule is also readily recognized and taken up by islet *β*-cells through the membrane glucose transporter GLUT2 into the intracellular compartment [16,17]. Elsner et al. [66] compared the uptake and toxicity of STZ and additional four structurally similar chemicals (MNU, ENU, MMS, and EMS) in bioengineered RINm5F insulin-producing cells. Results showed that cells expressing the GLUT2 transporter were far more susceptible to STZ’s cytotoxicity than the control cells, but GLUT2 expression had no similar effect on the toxicity of MNU, ENU, MMS, and EMS which were not taken up by GLUT2.

It is speculated that GLUT2 might be a selective target protein for covalent modification by STZ, and the modified GLUT2 might become an autoantigen, contributing to the development of T1DM. Somewhat in line with this hypothesis, it was reported earlier that there is a time-dependent reduction of GLUT2 protein level in animals treated with STZ, and the reduction occurs before the onset of hyperglycemia [17,67].

Here it is of note that in an earlier study [68], the viable rat islet cells were used to detect whether islet cell surface antibody (ICSA) was present in the sera of diabetic and control patients. It was found that ICSA was present in almost all recent-onset T1DM under 30 years of age (15/16); the occurrence was much higher than in normal controls (1/18) or in patients with autoimmune thyroiditis (1/12). Further analysis revealed that most recent-onset T1DM under 30 contained class I–ICSA, which is bound exclusively to islet *β*-cells. In addition, it was reported that this ICSA could also bind to human pancreatic islet cells [69]. It will be of considerable interest to determine whether the antigen present on rat and human islet cells to which ICSA binds is the GLUT2 protein that might be covalently modified by STZ or its breakdown products.

## 6. STZ Can Induce Cellular Immune Responses in Pancreatic Islets

Cellular immune responses are thought to play a major role in mediating persistent *β*-cell damage that eventually leads to the onset and pathogenesis of T1DM in humans [2,26]. From pancreatic biopsy of pre-diabetic patients or patients with new-onset T1DM, varying degrees of reduction in islet *β*-cell volume were observed in almost all cases, and insulitis was seen in about half of the cases studied [2,26]. In those samples where insulitis was observed, the cellular infiltrates were composed of CD8^+^ and CD4^+^ T cells, B lymphocytes, and macrophages, with a predominance of CD8^+^ T cells. The inflamed islet cells had elevated expression of MHC class I molecules [2,26]. Discussed below are some of the experimental observations showing that MLD-STZ treatment can also induce similar immunological changes in animal models of autoimmune T1DM.

### 6.1. Observations Made in Immune Function-Intact Laboratory Animals

In mice receiving MLD-STZ administration, necrotic changes and endothelial swelling were often seen in pancreatic islets. Histopathological analysis showed that CD4^+^ and CD8^+^ T cells as well as invading macrophages and MHC class II-positive cells were detected in the islets [70]. It was generally thought that the destruction of islet *β*-cells in animals receiving MLD-STZ administration was mediated by cellular immune response [60].

The role of cytokines in the progression of islet autoimmunity in MLD-STZ-induced diabetic mice had also been studied, which also reflected a cellular immune response against islet *β*-cells [2,52,71]. It was found that significant increases in Th1 cell-associated cytokines, such as IFN-*γ*, TNF-*α*, and IL-12 mRNA expression, were detected in the islets [52], and disrupting the production of IFN-*γ* or IL-12 could ameliorate DM development in these animals, suggesting a strong correlation between Th1 cell-mediated cellular immune response and pathogenesis of MLD-STZ-induced DM [2,71]. These observations were consistent with the results from other animal studies reporting that the dominant activation of Th1 over Th2 cells was a key determinant in the pathogenesis of MLD-STZ-induced autoimmune DM in rodents [52].

### 6.2. Observations Made in Immunodeficient Mice

In an earlier study, the T cell-deficient/athymic mice were used to investigate the role of cellular immune response in MLD-STZ-induced DM in rodent models [32]. It was observed that treatment of thymectomized BALB/c mice with MLD-STZ induced a significantly lower percentage of hyperglycemia (6/19) in T cell-deficient mice, whereas the control mice manifested a 100% incidence of hyperglycemia (10/10) [44]. These findings indicated that T cell-mediated immune response against chemically altered islet *β*-cells contributed importantly to the induction of DM in animals treated with MLD-STZ.

### 6.3. Insights Gained from Transplantation Studies

Researchers have studied the transfer of DM conditions through transferring spleen cells from STZ-treated mice to normal mice [44,56]. For instance, it was reported that transferred spleen cells obtained from DM mice (induced by MLD-STZ) to normal syngeneic mice failed to induce hyperglycemia in the recipients [44], but spleen cells transplanted to the syngeneic mice pretreated with a single low dose of STZ successfully induced a moderate but progressive increase in blood glucose levels (8/8). These observations indicated that pretreatment of recipient animals with STZ was necessary for the efficient transfer of DM conditions.

The pancreatic islet transplant model had also been used to explore the immunological mechanisms involved in MLD-STZ-induced DM [72]. It was found that insulitis in the syngeneic islet grafts and diabetes in the recipients occurred when the islet grafts were transplanted before the recipient animals were treated with MLD-STZ. On the contrary, when normal islet grafts were transplanted into the recipients after MLD-STZ administration, no inflammation was observed in the islet grafts, and the transplanted islets effectively prevented the development of hyperglycemia in the recipients [72]. Furthermore, STZ exposure of the islet grafts in vitro before transplantation also led to insulitis in the grafts after transplantation to recipients previously treated with STZ [72].

From these studies, it was clear that STZ exposure for both transplanted islets and recipients was a prerequisite for the autoimmune response to occur in the transplants. One logical explanation is that STZ exposure would modify islet *β*-cells immunologically, rendering them as newly-appeared autoantigens which can then be recognized and attacked by the body’s immune system.

## 7. STZ Can Induce the Production of Autoantibodies against Cellular Components of Pancreatic Islets

While persistent islet *β*-cell destruction in T1DM is thought to be mainly mediated by autoreactive T cells [2,26], the appearance of autoantibodies is actually the first detectable sign of *β*-cell autoimmunity and can be used for the prediction of T1DM in non-diabetic individuals [3,63]. A series of autoantigens have been identified in patients with T1DM. It has been suggested that cellular, as well as humoral immune responses, are both driven by autoantigens and regulated by various cytokines resulting in the pathogenesis of autoimmune T1DM.

### 7.1. Clinically Identified T1DM-Related Autoantibodies

The presence of T1DM-associated autoantibodies is a clinical characteristic of T1DM, and their appearance would enable the prediction of disease occurrence well before clinical symptoms arise [3]. Highly sensitive laboratory measurements can capture 98% of individuals with autoantibodies at diagnosis. The main ones associated with autoimmune T1DM development include islet cell autoantibodies (ICA), insulin autoantibodies (IAA), autoantibodies targeting the glutamic acid decarboxylase (GADA), autoantibodies targeting the tyrosine phosphatase-related insulinoma-associated 2 molecules (ICA512 or IA2A), zinc transporter-8 autoantibodies (ZnT8), and tetraspanin-7 autoantibodies (reviewed in [3,63,73]).

One or more autoantibodies are usually detected at different stages of DM pathogenesis before its clinical onset [74]. The number of detected autoantibodies is related to the risk of clinical onset. Research has shown that the largest increase in risk was associated with the presence of two or more autoantibodies [74]. Children who were tested positive for two or more of the aforementioned autoantibodies were at an increased risk (50–100%) for developing T1DM over the next 5–10 years [3,75].

Clinical studies showed that autoantibodies might occur as early as 3 months of age [74,75,76,77]. A greater percentage of children develop autoantibodies between the ages of 9 months and 3 years, and the first autoantibody appearing in infants is usually IAA, with GADA being the second most frequent autoantibody [74,75,76,77]. GADA is more common than IAA in *HLA-DR3/3* children but less common in *HLA-DR4/8* children [74]. In addition, it has been reported that children with T1DM have increased titers of autoantibodies against SS-DNA, Poly A-U, and Poly I-C compared to children in the control group [78]. The presence of SS-DNA autoantibodies in diabetic children indicates the autoimmune nature of the disease.

It has been suggested that T1DM most likely is triggered at an early age by autoantibodies primarily directed against cellular components in islet *β*-cells [3,26,47]. After the initial appearance of one of these autoantibody biomarkers, a second, third, or fourth autoantibody against other cellular components might also appear. The larger the number of *β*-cell autoantibody types, the greater the risk of rapid progression to clinical onset of diabetes. This association does not necessarily mean that the *β*-cell autoantibodies are pathogenic, but rather they represent reproducible biomarkers of the autoimmune-mediated pathogenic process. Based on clinical observations of autoantibodies in young children, it has been suggested that positivity for a single autoantibody usually would represent a harmless non-progressive *β*-cell autoimmunity in most cases, whereas the presence of two or more autoantibodies would reflect a progressive process of the disease [3]. It is of note that the development of islet *β*-cell autoimmunity clearly involves a genetic risk factor as the pathogenesis of DM often occurs in individuals with the *HLA-DR3-DQ2* and/or *HLA-DR4-DQ8* haplotypes [79,80,81]. The role of genetic factors in the pathogenesis of T1DM is separately discussed in Section 8.2.

### 7.2. Antibodies Identified in STZ-Induced Diabetic Animal Models

DM-associated autoantibodies were also found in STZ-induced diabetic animals. It had been reported that there was significant mononuclear cell infiltration in pancreatic islets of CD-1 mice at 21 days after receiving STZ treatment [82]. Immunoglobulin deposition in islets was also observed in the majority (23/30) of the mice [82]. Further analysis showed that 17 of the mice had solely IgG deposition while 6 had either IgM or IgG in separate islet tissues. The detected circulating antibodies included poly A:U (6/30), poly I:C (11/30), single-stranded DNA (23/30), and anti-nuclear autoantibodies (10/30) [82]. The observations reported in this study were quite similar to the islet cell autoantibodies found in humans. It will be of considerable interest to determine whether some of the clinically observed T1DM-associated autoantibodies are related to the cellular components covalently modified by STZ or its breakdown products.

## 8. Environmental and Genetic Factors Affecting the Development of T1DM

Many years ago, a model was proposed to explain the etiology and development of T1DM, which speculated that people are born with varying degrees of genetic susceptibility to T1DM, and the disease is only triggered when a genetically susceptible individual is exposed to a conducive environmental risk factor(s) as a trigger [9]. In the proposed STZ-induced T1DM pathogenesis model, genetic predisposition/susceptibility and the environmental risk factor (trigger) are critical pathogenic components, and in the absence of these necessary conditions, STZ alone likely would not be sufficient to induce overt autoimmune T1DM in many human cases. Provided below is a brief discussion of the environmental and genetic factors affecting the pathogenesis of autoimmune T1DM.

### 8.1. Environmental Factors

As aforementioned, a long list of environmental risk factors has been suggested to be potentially involved in the pathogenesis of T1DM [10]. Among these factors, viral infection is an important causative factor [83]. It is speculated that an environmental insult, often in the form of severe febrile viral infections, is involved in initiating the pathogenic process in genetically susceptible individuals. This external influence likely would precipitate an inflammatory response in pancreatic islets known as insulitis which is often accompanied by infiltration of activated T-lymphocytes.

Offering partial support for this idea, there was a large prospective cohort study reporting a strong association between early-life respiratory infections and later clinical T1DM [84]. It was found that among young children (during their first 4 years of life), the number of respiratory infection episodes (RIEs) within a given 9-month period was associated with the subsequent onset of islet autoimmunity in the ensuing 3 months. For each 1-per-year rate increase in infections, the hazard of islet autoimmunity was increased by 5.6%. The types of respiratory infections independently associated with autoimmunity are common cold, influenza-like illness, sinusitis, and laryngitis/tracheitis. The RIE-associated risk of islet autoimmunity was observed primarily for winter RIEs, which is the high season for respiratory infections. The infection types observed in the winter season are mostly caused by different respiratory viruses, although bacteria are also frequently involved in sinusitis [84].

It is of note that febrile RIEs represent a more consistent risk association with islet autoimmunity compared with non-febrile RIEs, suggesting that fever may be a factor that increases the islet autoimmunity risk [85]. The clinical course of febrile infections tends to be more severe and is often associated with pronounced viremia that allows the spread of the virus to the pancreas. It was suggested that severe infections are also associated with heightened stress in the endoplasmic reticulum of islet *β*-cells [82], which might alter post-translational modifications of the *β*-cell autoantigens, likely making them more immunogenic and thus promoting autoimmunization [86,87].

In addition to respiratory viruses, enteroviruses have also been suggested to be another important factor that triggers islet autoimmunity in the body [88,89]. Studies in animal models and human subjects have indicated that enterovirus infections during early infancy were associated with increased risk for *β*-cell autoimmunity [10,90]. A large prospective study has found that exposure to enterovirus infections either in utero or during childhood might initiate *β*-cell damage and subsequent T1DM.

In the proposed STZ-induced pathogenesis of human T1DM, environmental risk factors play a critical role. It is speculated that severe febrile viral infections, caused either by respiratory viruses or enteroviruses, are important risk factors that might trigger the body’s aberrant immune responses against STZ-exposed islet cells in certain genetically susceptible individuals. In the absence of the environmental trigger(s), the body likely may not develop overt islet autoimmunity even in genetically predisposed individuals.

### 8.2. Genetic Factors

It has long been suspected that T1DM has an important genetic susceptibility component involved [91,92]. For instance, it is known that T1DM has an identical twin concordance of 30–70% [93]. Two HLA class II haplotypes are involved in antigen presentation, namely, HLA DRB1*0301-DQA1*0501-DQ*B10201 (*DR3*) and HLA DRB1*0401-DQA1*0301-DQB1*0301 (*DR4-DQ8*), are linked to approximately 50% of disease heritability and are prevalent in white people [94,95]. Certain haplotypes are known to reduce T1DM risk, such as DRB1*1501-DQA1*0102-DQB1-0602 (*DR15-DQ6*) [89]. In addition, genome-wide association studies have identified over 60 additional non-HLA loci associated with the risk of T1DM [2,94,96].

Animal experiments have also shown that mutations of genes in certain susceptibility loci including *Il2*, *Il2ra* (*CD25*), *Ctla4*, *PTPN22*, and *Pdca1* (*PD-1*) significantly increase the susceptibility for a number of autoimmune diseases, including T1DM [1].

In the proposed hypothesis on the pathogenesis of autoimmune T1DM, the STZ-induced autoimmunity in islet *β*-cells would be favored to occur when the body’s immune system is activated in an aberrant fashion, which is critically aided by certain genetic factors. However, it is presently unclear how these genetic risk factors (such as the *HLA* haplotypes) cooperate with critical environmental factors to alter the risk for developing STZ-induced autoimmune attacks against islet *β*-cells.

## 9. Other Potential Factors Related to T1DM

### 9.1. Potential Role of Gut Microbiota in T1DM

As mentioned earlier, there are about 500 to 1000 species of gut microorganisms in humans [28]. Studies have indicated that gut microbiota is associated with T1DM development [29,30,31]. It was observed that in Bio-Breeding DB-prone rats, the composition of gut microbiota before the onset of T1DM was markedly different between the rats that eventually would or would not develop T1DM [29,97]. Gut microbial composition is also different between T1DM patients and healthy human subjects [95,96]. It appears that the microbiota of healthy children is more diverse and stable compared with children who eventually develop T1DM [98,99,100]. An earlier study suggested that diet could alter the gut microbial composition and affect T1DM development in humans and animal models [101]. Moreover, it was reported that modulation of gut microbiota in Bio-Breeding DM-prone rats through administration of antibiotics decreased the incidence and also delayed the onset of DM [29,31].

It is speculated that certain bacteria capable of producing STZ might be present in human gut microbiota, and if this is indeed the case, then it is likely that those individuals who are more prone to develop T1DM might have a higher population of STZ-producing bacteria in their gut microbiota. It will be of interest to experimentally examine this intriguing possibility.

### 9.2. Potential Relationship between Cow’s Milk Feeding and Autoimmune T1DM

Some epidemiological studies have speculated that early dietary intake of cow’s milk proteins significantly increased the risk of *β*-cell autoimmunity and T1DM, especially in children with *HLA-DQB1* genotypes [33,34]. Similarly, there were also studies reporting a possible correlation between antibodies against cow’s milk proteins (e.g., bovine serum albumin BSA) and the incidence of autoimmune T1DM. For instance, the Finland Study Group examined the humoral immune response to cow’s milk proteins in pediatric patients with newly diagnosed T1DM [102]. It was found that the IgA and IgG antibodies against BSA and *β*-lactoglobulin were higher in T1DM patients compared with their non-diabetic siblings as well as normal controls, indicating a stronger humoral response to cow’s milk proteins occurring in young T1DM patients [88]. Similarly, Levy-Marchal et al. [103] found elevated anti-BSA antibodies in 74.4% of newly diagnosed T1DM children, significantly higher than those observed in ICA-positive non-diabetic subjects and normal controls. In addition, associations of enhanced immune response to two other cow’s milk proteins, *β*-lactoglobulin and *β*-casein, were observed in pediatric T1DM [104,105]. These results indicate that some genetically susceptible children who have an increased intake of cow’s milk products might be associated with an increased risk for developing diabetic autoimmunity. Somewhat in line with the above speculation, an earlier meta-analysis reported that breastfeeding had some, although limited, protection against T1DM development [106]. Further, the duration of breastfeeding and the age of introduction of cow’s milk formula are inversely associated with T1DM risk in a clear dose-dependent manner [107]. However, it should also be clearly noted here that the above speculation has been highly controversial in the past, as there were also many investigators who would disagree with it [108,109].

As mentioned earlier, animal fodders and other organic materials are habitats for the STZ-producing bacteria *Streptomyces* [15]. Animal fodders are usually comprised of hay, straw, silage, compressed and pelleted feeds, oils, mixed rations, sprouted grains, and legumes (such as bean sprouts, fresh malt, or spent malt). It is possible that cows might ingest a certain amount of *Streptomyces* and other bacteria from their fodders that might be able to produce STZ, and as such, STZ might be present in cow’s milk and its products. It is also possible that the STZ-producing bacteria *Streptomyces* might be present in cow’s gut microbiota. In either case, it is potentially possible that early dietary intake of cow’s milk products may increase the intake of free STZ in young children, and if that were the case, then it might lead to increased exposure to islet *β*-cells to STZ and thus enhance STZ-induced autoimmunity. In addition, STZ contained in cow’s milk products may form STZ-cow milk protein conjugates. Their formation would serve as immunogens and may facilitate the formation of antigen-specific antibodies in the body. While some of these antibodies likely would readily react with cow’s milk proteins, some others might also cross-react with STZ-conjugated macromolecular components contained in islet β cells. As such, early childhood use of cow’s milk products might be associated with an increased risk for developing diabetic autoimmunity. Future laboratory studies are needed to carefully and critically examine these potential possibilities.

### 9.3. A Potential Strategy for Reducing the Risk of Developing T1DM

If STZ is confirmed in the future to be an important causative factor in human autoimmune T1DM, then it is apparent that it would be highly beneficial to supply the body with more *α*-*D*-glucose (in the form of saline glucose solution) during severe febrile infections (pediatric patients in particular) as the glucose molecule is a competitive inhibitor of GLUT2-mediated STZ transport and thus would help reduce the risk of developing T1DM. In support of this notion, animal studies have shown that pre-injection of mice with *α*-*D*-glucose and 5-thio-*D*-glucose before STZ administration could effectively abrogate STZ-induced reduction of GLUT2 protein levels as well as hyperglycemia [110,111].

## 10. Concluding Remarks

STZ, originally identified in the late 1950s as an antibiotic [11], was later found to be preferentially toxic to the insulin-producing islet *β*-cells [16,17] as it could be selectively transported by GLUT2 into these cells. It is hypothesized that humans might be occasionally exposed to STZ through opportunistic infections with the STZ-producing bacteria and/or through ingestion of certain food products that contain STZ. In addition, the possible presence of STZ-producing bacteria in human gut microbiota may also increase the likelihood of long-term STZ exposure. Because of the high chemical reactivity of STZ and its breakdown products, these chemicals, when present inside human islet *β*-cells, would covalently modify certain cellular macromolecules (e.g., DNA and proteins), and the covalently modified macromolecules may serve as new autoantigens with the potential to induce humoral and cellular autoimmune responses in pancreatic islets of genetically susceptible individuals. The chances for the eventual development of autoimmunity against STZ-exposed islet *β*-cells likely would be significantly increased if the body is concurrently having strong aberrant immune responses stimulated by severe febrile viral infections, which likely would serve as a critical environmental trigger for the pathogenic process.

While the proposed hypothesis is partially supported by some direct or indirect (circumstantial) evidence from laboratory and clinical studies, there are also important unanswered questions. For instance, it is known that GLUT2 is present in the liver and intestine [112,113,114], in addition to islet *β*-cells [16,17]. Would cells in these sites also suffer similar autoimmune-type damage like islet *β*-cells? The answer is quite clear based on clinical observations: people with autoimmune T1DM usually do not develop concurrent autoimmune liver or intestinal diseases. There might be explanations for the apparent target site preference of STZ’s pathogenicity. When STZ is present inside the human body, it is expected that this chemical would also exert certain levels of direct cytotoxicity in those cells (such as hepatocytes and intestinal epithelial cells) that express GLUT2. However, since hepatocytes and intestinal epithelial cells usually express very high levels of xenobiotic-metabolizing enzymes (including both Phase I and II metabolizing enzymes) [115], it is expected that the rate of metabolic disposition of STZ in these target sites would be much faster than in other cells such as islet *β*-cells. In addition, hepatocytes usually contain high levels of glutathione [116,117], which would be an important protective factor in neutralizing the chemically reactive STZ and its breakdown products. Therefore, it is likely that hepatocytes may suffer significantly less damage compared to islet *β*-cells when human subjects are exposed to STZ. This suggestion appears to be in line with some of the clinical observations [18,118,119]. For instance, clinical studies have shown that when STZ was given to patients with islet cell carcinoma, strong cytotoxicity was seen in the pancreatic islets, whereas toxicity in the liver and gastrointestinal tract was observed but less severe [18,118,119]. Notably, similar observations were also made in experimental studies using laboratory animals [21,46]. It was found that when the animals were treated with STZ, there was preferential damage to islet *β*-cells, whereas damage to other organs, including the liver and intestine, was relatively mild [21,46].

As aforementioned, STZ can readily cause covalent modifications of cellular macromolecules which may result in the formation of new antigens. It is hypothesized that the development of autoimmune responses against STZ-exposed target cells (such as islet *β*-cells) would only occur in genetically susceptible individuals in the joint presence of a strong environmental trigger. When these critical genetic and environmental factors are absent, autoimmune responses may not take place, even if the islet *β*-cells are already covalently modified by STZ or its breakdown products to form new cellular autoantigens. This is probably also the reason why most patients with islet cell carcinoma who received STZ-based chemotherapy did not develop overt autoimmune DM afterward [18,118,119]. Similarly, it is speculated that while many people in the general population might be exposed to varying levels of STZ (either through environmental sources or resulting from the endogenous production by their own gut microbiota), only a very small fraction of people eventually would develop T1DM. This discrepancy might highlight the critical importance of the genetic and environmental factors working together to bring about the “perfect storm” of an aberrant autoimmune attack against STZ-exposed islet *β*-cells. It is likely that these genetic and environmental factors suitable for the induction of islet *β*-cell autoimmunity may not work equally well for other organs such as the liver and intestine. It is certain that more experimental studies are needed to carefully and critically examine each of the key elements associated with the proposed pathogenic hypothesis on human autoimmune T1DM, and it would be fascinating to decipher the mechanisms underlying the complex interplays between genetic factors and environmental triggers that ultimately help bring about the pathogenic autoimmune attack against STZ-exposed islet *β*-cells.

## Data Availability

Not applicable.
